# Analyzing the use of specialized palliative care in intensive care unit patients in Germany: a cross-sectional study

**DOI:** 10.1186/s12904-025-01718-1

**Published:** 2025-03-20

**Authors:** Christiane von Saß, Theresa Tenge, Birgitt van Oorschot, Dawid Pieper, Nicole Eisenmenger, Martin Heinze, Larissa Fink, Guido Michels, Martin Neukirchen, Marcel A. Kamp

**Affiliations:** 1 Palliative Care, Brandenburg Medical School Theodor Fontane and Faculty of Health Sciences Brandenburg, Rüdersdorf bei, Berlin Germany; 2 Interdisciplinary Centre for Palliative Care, Medical Faculty and University Hospital Düsseldorf, Heinrich-Heine-University, Düsseldorf, Center for Integrated Oncology Aachen Bonn Cologne Düsseldorf (CIO ABCD), Düsseldorf, Germany; 3 Department of Anesthesiology, Medical Faculty, University Hospital Duesseldorf, Heinrich-Heine-University, Düsseldorf, Germany; 4 Interdisciplinary Center for Palliative Medicine, Clinic for Radiation Oncology, University Hospital Würzburg, Würzburg, Germany; 5 Faculty of Health Sciences Brandenburg, Brandenburg Medical School Theodor Fontane, Institute for Health Services and Health System Research, Rüdersdorf, Germany; 6 Center for Health Services Research, Brandenburg Medical School Theodor Fontane, Rüdersdorf, Germany; 7 Reimbursement Institute, Hürth, Germany; 8 Center for Mental Health, Immanuel Clinic Rüdersdorf, University Hospital of Brandenburg Medical School Theodor Fontane, Rüdersdorf bei, Berlin Germany; 9 Department of Emergency Medicine, Hospital of the Barmherzige Brüder, Trier, Germany; 10 Immanuel Clinic Rüdersdorf, University Hospital of the Brandenburg Medical School Theodor Fontane, Rüdersdorf bei, Berlin Germany

**Keywords:** Palliative medicine, Critical care, Interdisciplinary research, Long-term ventilation, Age, Gender, Mortality

## Abstract

**Background:**

Despite rising importance of integration of palliative medicine in treating life-threatening illnesses in intensive care units (ICU), the extent remains unknown. Using billing data, we analysed the frequency of specialized palliative care use in ICU patients in Germany.

**Methods:**

Billing data (2019 -2022) from the InEK was used in this cross-sectional study on all billed adult ICU cases. Data included case numbers, demographics, diagnoses, treatment procedures, ventilation (≥ 95 h), palliative care frequency.

**Results:**

61,591,299 adult cases were treated, 11.2% (6,912,316) requiring ICU and 499,262 (7.2%) needing long-term ventilation. 44.2% of all ICU cases and 36.2% of long-term ventilated patients were female (*p* < 0.0001). ICU mortality was 11.1%, long-term ventilation mortality was 38.8%; higher in men and patients aged ≥ 65 (*p* < 0.001). Leading diagnoses for ICU deaths: heart failure (6.9%), stroke (6.3%), sepsis (6.2%).

0.8% of ICU cases and 1.4% of long-term ventilated cases received specialized palliative care, with a higher proportion of females (*p* < 0.0001). Most palliative care patients were aged ≥ 65.

**Conclusion:**

From 2019 to 2022, 11.2% of hospital cases required ICU-treatment. Despite suffering from life-threatening conditions and high mortality rates, less than 1% of all ICU cases and 1.4% of long-term ventilated cases received palliative care (differing sexes and ages). This highlights deficiencies in palliative care integration into ICUs to alleviate patients and their families suffering from complex needs. Implementing benchmarking could be beneficial in this process.

**Supplementary Information:**

The online version contains supplementary material available at 10.1186/s12904-025-01718-1.

## Introduction

Currently, there are compelling discussions surrounding the management of intensive care patients and their access to palliative care [1]. Germany has one of the highest densities of intensive care beds worldwide. However, no standardized criteria exist for inpatient intensive care treatment. ICU patient populations are highly diverse, ranging from critically ill individuals requiring mechanical ventilation or other life-saving organ-support therapies to those needing postoperative or extended monitoring [2]. ICU structures also vary, operating as either multidisciplinary or department-specific units. Multidisciplinary ICUs manage patients from multiple specialties, often with significant involvement from anesthesiologists, while department-specific ICUs focus on fields such as surgery, internal medicine, cardiology, neurology, or infectious diseases. The organization and specialization of ICUs differ across hospitals based on size, resources, and medical focus.

Intensive care units (ICUs) typically cater to patients with potentially life-threatening conditions and a limited prognosis. These patients, along with their families, often experience a significant symptom burden, including physical, psychological, social, spiritual and communication challenges [3–8]. Palliative care aims to address the needs of patients facing life-threatening illnesses and their families. Despite the evident overlap between palliative and intensive care medicine, the concept of integrated palliative and intensive care only began to emerge in the late 1990s [9]. While intensive care treatment focusses on prolonging life through extensive therapy, palliative medicine prioritizes enhancing quality of life with minimal technology use. Nevertheless, both disciplines complement each other [10]. Today, palliative care principles, including thorough symptom management and clear communication, have become integral components of corresponding intensive care recommendations [11, 12]. Moreover, various medical societies and guidelines recommend an early integration of palliative care for seriously ill and ICU patients [11, 13, 14]. In Germany, a recent position paper underscores the importance of timely integration of palliative care in clinical acute, emergency, and intensive care medicine and aims to enhance patients' quality of life and alleviate symptoms [12]. The German Interdisciplinary Association for Intensive Care and Emergency Medicine (DIVI) recommends integrating palliative care into intensive care units and ensuring the availability of simultaneous intensive and palliative care treatments [15].

In Germany, primary care physicians and nurses with basic palliative care training oversee general palliative care. In contrast, specialized inpatient palliative care is delivered by multiprofessional teams. These teams offer consultation services to general and ICU wards for patients with complex palliative care needs or palliative care treatment on a palliative care ward. Reliable international and German data on the integration of palliative and intensive care medicine are limited. In the mid-2010s, about a quarter of all hospital deaths and nearly 12% of all deaths in Germany occurred in an ICU [16].

A 2017 survey at German Comprehensive Cancer Centers found that 11 out of 15 centers had palliative medicine consultation services, caring for a median of 33 ICU patients and admitting a median of 9 patients to palliative wards annually. Two centers had regular visits from both ICU and palliative care teams [17]. Currently, no data exists on the number of cases involving concurrent palliative and intensive care treatment in Germany. The aim of the present study was to determine the frequency of specialized palliative care treatment in hospitalized patients admitted to German ICUs.

## Methods

### Study design

In this cross-sectional study, we evaluated data sourced from the Institute for the Remuneration System in the Hospital Sector (InEK GmbH, Siegburg, Germany) covering the period from 01.01.2019 to 31.12.2022. We exclusively relied on public data obtained after approval by the institutional and local ethics committee (study ID: 190032024-ANF, ethics committee of the Brandenburg Medical School, Germany). We followed the STROBE statement (Suppl. Table 3) [18].

### Setting and data source

In Germany, all hospitals are reimbursed for their services using a performance-based, flat-rate remuneration system (§ 17b, *Krankenhausfinanzierungsgesetz*, Hospital Financing Act) based on the German Diagnosis Related Groups System (G-DRG). A specific DRG rate is assigned to each inpatient treatment case for reimbursement. It is compulsory for all German Hospitals to send their data (demographics, primary and secondary diagnoses, procedures) to InEK GmbH. InEK GmbH has been legally delegated to implement and maintain this system and aggregate received data making it publicly available via the InEK Browser (§21 Hospital Fees Act).

### Cohort / Participants

Data for all billed hospital cases involving ICU care meeting the following criteria was obtained:

1. > 18 years of age
2. total number of hospital cases,
3. total number involving ICU care
4. total number of ICU cases and those ICU cases with > 95 h ventilation

### Variables and definitions

We retrieved billing data for German hospital cases and analyzed the number of cases coded per specific code. Thus, our data represent the number of hospital cases in which the respective code was assigned. Diagnoses were classified according to the International Statistical Classification of Diseases and Related Health Problems, 10th Revision, German Modification (ICD-10-GM). In the German DRG system, each case has a single primary diagnosis and may include multiple secondary diagnoses. Medical procedures and treatments were identified using their corresponding procedural codes (*Operationen- und Prozedurenschlüssel, OPS*). Data extracted included:

1. case numbers for each cohort
2. demographics (age groups as predefined by InEK browser, sex distribution classified into female, male, diverse, unknown)
3. case distribution among hospitals according to bed capacity and ownership
4. count of primary diagnoses and treatment procedures
5. Administration of specialized palliative care according to Operations and Procedure Codes (OPS 8–982, 8-98e, 8-98 h) and complex intensive care treatment (OPS 8–980, 8-98f, for code definition see Supplemental Table 1).

Intensive care cases were identified using the InEK data browser, applying the selection criteria for intensive care cases. This approach includes all German ICU cases, regardless of the specialized department. The browser also allows for selecting cases based on ventilation duration. Long-term ventilation was defined according to the G-DRG system as mechanical ventilation lasting more than 95 h in combination with intensive care complex treatment [19].

### Bias

We minimized selection bias by including all consecutive adult ICU admissions in Germany from 2019 to 2022, ensuring a comprehensive and representative sample. However, our analysis pertains to hospital cases rather than individual patients. The distinction between hospital and patient cases can introduce bias when assessing the frequency of specialized palliative care for a specific illness. Patients may have multiple hospitalizations but receive palliative care only once. In our study, this distinction is less relevant, as we analyzed the co-occurrence of intensive care and specialized palliative care within the same hospitalization. Moreover, not all ICU patients have life-threatening conditions, such as those admitted for monitoring after planned surgery. Therefore, we focused on ICU cases with ventilation exceeding 95 h, as these patients inherently face life-threatening conditions. Reported data is complete considering all billed cases reported to and made available by the InEK from all German Hospitals with the exception of potential results of less than four cases for data protection. Considering the high volume of cases analyzed, this small number of cases should not affect the overall outcome. To reduce measurement bias, we extracted data on palliative care consultations and intensive care treatments using predefined OPS and ICD-10 codes, ensuring reliable and consistent identification of in-patient ICU cases receiving specialized palliative care. While OPS and ICD-10 classifications are well established, we cannot entirely rule out misclassifications. Measurement errors are minimal in our study based on billing data. The study aimed to determine the frequency of palliative medical treatment during ICU stays and long-term ventilation, using descriptive statistics, which minimizes the impact of confounding bias. However, information on key demographic and clinical variables, such as age, gender, and severity of illness, are given in detail.

### Statistics

Data were obtained from the InEK data browser and organized using Microsoft Excel for Mac (version 16.78, Microsoft Corporation, Redmond, Washington, USA). Statistical analyses and graphical representations were conducted using GraphPad Prism 9 for macOS (version 9.5.0, GraphPad Software, Inc., La Jolla, USA).

Descriptive statistics were utilized to calculate the percentages of patients who passed away and those who received treatment. The Chi-square test with Yates' correction was employed to examine differences in sex distributions across various subgroups [20]. A two-sided significance level of α = 0.05 was applied, with a Bonferroni correction for multiple comparisons (*n* = 8) [21]. Thus, the adjusted significance level was set at 0.006. To assess the association between two categorical variables in 2 × 2 contingency tables, we calculated odds ratios (OR) along with their corresponding 95% confidence intervals (95%-CI) [22].

## Results

### Patient cohorts and baseline characteristics

Between 2019 and 2022, Germany had 61,591,299 adult hospital cases. Among these, patients required intensive care in 6,912,316 hospital cases (11.2%), and ventilation > 95 h in 499,262 cases (7.2% of ICU cases). A complex or specialized ICU therapy (OPS codes 8–980 or 8-98f) was conducted and billed in 2,485,363 ICU cases (35.9%) and in 467,681 ICU cases with long-term ventilation (93.7% of these; Supplement Figs. 1 and 2).

**Fig. 1:**
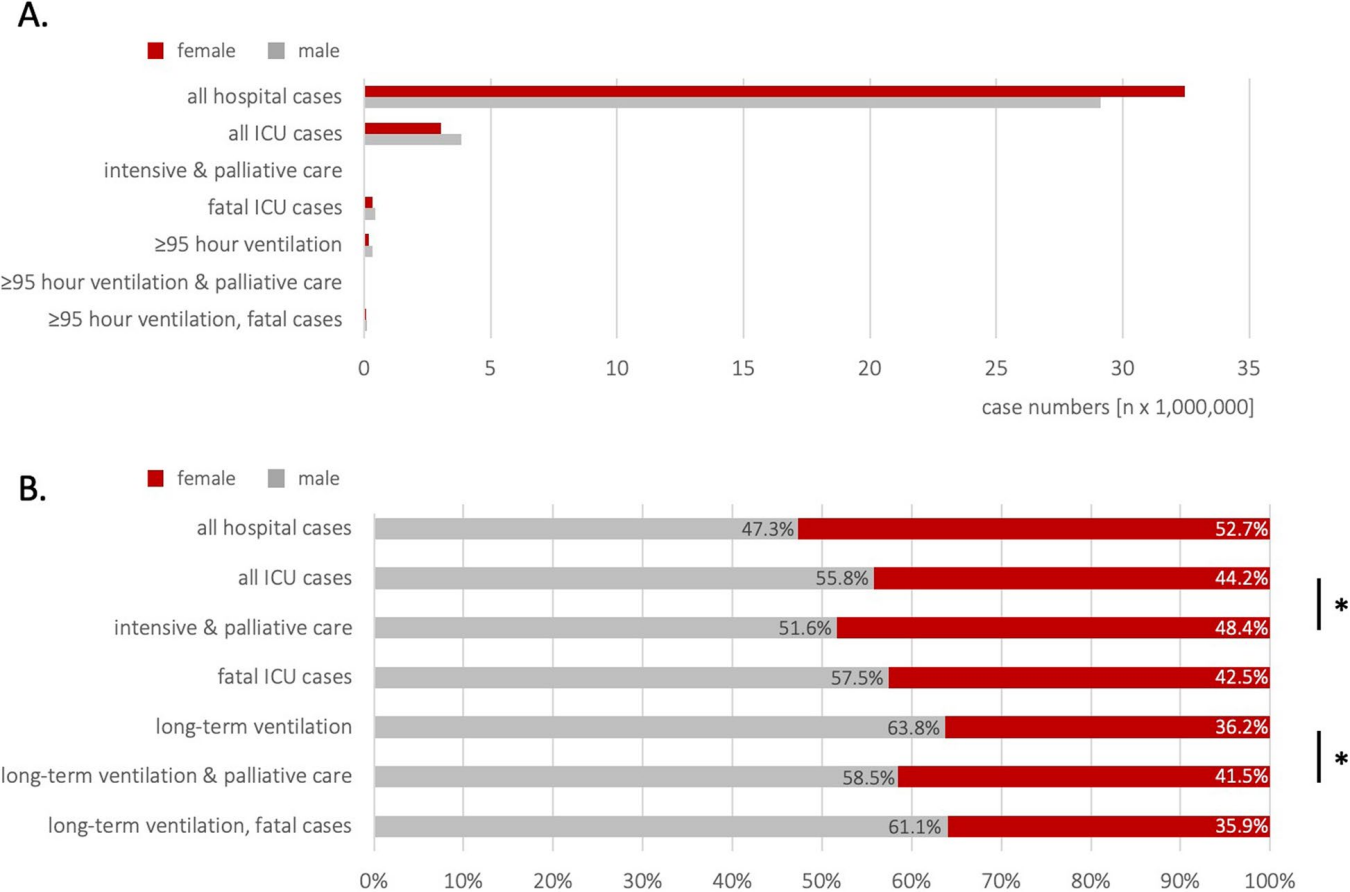
Sex distribution. The figure illustrates the gender distribution in the analysed hospital case cohorts. Notably, the proportion of cases with female patients receiving palliative care was significantly higher. Once again, the proportion of cases with female patients in long-term ventilated cases receiving palliative care was significantly higher than in the comparison group. *Demonstrates a significant difference (*P* < 0.006)

Female patients accounted for 52.7% of all hospital cases (32,449,759 cases) but only 44.2% of ICU cases (3,054,198 patients) and 36.2% of long-term ventilation cases (180,799 cases; *p* < 0.0001, Table 1 and Fig. 1). The age distribution also varied significantly: whilst 26.7% of all hospital patients were ≥ 65 (32,252,121 cases), 63.3% of ICU patients and 61.8% of long-term ventilated patients (*p* < 0.0001) were older than 65 years.

**Table 1:** The sex and age distribution of the patients

	**2022**		**2021**		**2020**		**2019**		**2019–2022**	
**All hospital cases in Germany**										
Case numbers	14,770,158		14,761,106		14,906,432		17,153,603		61,591,299	
**Gender**										
Male	7,012,466	47.5%	6,964,983	47.18%	7,069,275	47.42%	8,091,024	47.17%	29,137,749	47.3%
Female	7,756,869	52.5%	7,795,307	52.81%	7,836,419	52.57%	9,061,164	52.82%	32,449,761	52.7%
Diverse	224	0.0%	155	0.00%	103	0.00%	24	0.00%	506	0.0%
Unknown	599	0.0%	661	0.00%	635	0.00%	1391	0.01%	3286	0.0%
**Age groups**										
18–29 years	1,050,691	7.1%	1,088,778	7.38%	1,109,110	7.44%	1,338,137	7.80%	4,586,716	7.4%
30–39 years	1,336,137	9.0%	1,402,848	9.50%	1,378,709	9.25%	1,538,006	8.97%	5,655,700	9.2%
40–49 years	1,079,507	7.3%	1,127,082	7.64%	1,142,259	7.66%	1,352,614	7.89%	4,701,462	7.6%
50–54 years	848,133	5.7%	913,708	6.19%	953,138	6.39%	1,133,731	6.61%	3,848,710	6.2%
55–59 years	1,192,188	8.1%	1,221,518	8.28%	1,234,549	8.28%	1,400,423	8.16%	5,048,678	8.2%
60–64 years	1,363,402	9.2%	1,333,806	9.04%	1,323,477	8.88%	1,477,227	8.61%	5,497,912	8.9%
65–74 years	2,904,668	19.7%	2,804,386	19.00%	2,752,010	18.46%	3,097,215	18.06%	11,558,280	18.8%
75–79 years	1,422,228	9.6%	1,462,512	9.91%	1,662,066	11.15%	2,092,452	12.20%	6,639,258	10.8%
80 + years	3,573,204	24.2%	3,406,468	23.08%	3,351,114	22.48%	3,723,798	21.71%	14,054,585	22.8%
**All ICU patients in Germany**										
Case numbers	1,706,703		1,736,413		1,818,032		1,651,168		6,912,316	
**Gender**										
Male	952,014	55.8%	969,951	55.86%	1,015,880	55.9%	919,834	55.71%	3,857,681	55.8%
Female	754,578	44.2%	766,354	44.13%	802,063	44.1%	731,203	44.28%	3,054,199	44.2%
Diverse	25	0.0%	17	0.00%	8	0.0%	5	0.00%	55	0.0%
Unknown	86	0.0%	91	0.01%	81	0.0%	126	0.01%	384	0.0%
**Age groups**										
18–29 years	57,178	3.4%	3.28%	56,888	3.28%	59,602	62,589	3.79%	236,257	3.4%
30–39 years	67,655	4.0%	3.98%	69,144	3.99%	72,493	68,982	4.18%	278,274	4.0%
40–49 years	94,954	5.6%	5.81%	100,969	5.79%	105,295	98,088	5.94%	399,306	5.8%
50–54 years	88,661	5.2%	5.57%	96,787	5.70%	103,710	96,808	5.86%	385,966	5.6%
55–59 years	135,555	7.9%	8.20%	142,361	8.17%	148,467	133,821	8.10%	560,204	8.1%
60–64 years	171,201	10.0%	9.92%	172,192	9.66%	175,566	155,123	9.39%	674,082	9.8%
65–74 years	398,794	23.4%	22.85%	396,723	21.97%	399,469	356,548	21.59%	1,551,535	22.4%
75–79 years	199,663	11.7%	12.25%	212,782	13.75%	250,019	247,338	14.98%	909,802	13.2%
80 + years	493,042	28.9%	28.14%	488,567	27.69%	503,411	431,871	26.16%	1,916,892	27.7%
**ICU patients receiving > 95 h ventilation**										
Case numbers	118,485		141,175		127,142		112,460		499,262	
**Gender**										
Male	75,335	63.6%	90,551	64.14%	81,550	64.14%	70,982	63.12%	318,418	63.8%
Female	43,134	36.4%	50,612	35.85%	45,581	35.85%	41,472	36.88%	180,799	36.2%
Diverse	2	0.0%	0	0.00%	1	0.00%	0	0.00%	3	0.0%
Unknown	14	0.0%	12	0.01%	10	0.01%	6	0.01%	42	0.0%
**Age groups**										
18–29 years	2189	1.8%	2179	1.54%	2050	1.61%	1925	1.71%	8343	1.7%
30–39 years	3571	3.0%	4062	2.88%	3481	2.74%	2862	2.54%	13,976	2.8%
40–49 years	6519	5.5%	8448	5.98%	6832	5.37%	5781	5.14%	27,580	5.5%
50–54 years	6905	5.8%	8977	6.36%	7808	6.14%	6758	6.01%	30,448	6.1%
55–59 years	11,254	9.5%	13,956	9.89%	11,903	9.36%	10,480	9.32%	47,593	9.5%
60–64 years	15,433	13.0%	18,398	13.03%	15,442	12.15%	13,343	11.86%	62,616	12.5%
65–74 years	35,963	30.4%	41,927	29.70%	36,293	28.55%	31,216	27.76%	145,399	29.1%
75–79 years	14,987	12.6%	18,716	13.26%	19,453	15.30%	19,024	16.92%	72,180	14.5%
80 + years	21,664	18.3%	24,512	17.36%	23,880	18.78%	21,071	18.74%	91,127	18.3%
**Deceased ICU patients**										
Case numbers	198,387		204,851		194,049		172,749		770,036	
**Gender**										
Male	113,665	57.3%	119,094	58.14%	111,750	57.59%	97,837	56.64%	442,346	57.4%
Female	84,672	42.7%	85,714	41.84%	82,273	42.40%	74,897	43.36%	327,556	42.5%
Diverse	3	0.0%	1	0.00%	2	0.00%	0	0.00%	6	0.0%
Unknown	47	0.0%	42	0.02%	24	0.01%	15	0.01%	128	0.0%
**Age groups**										
18–29 years	933	0.5%	828	0.40%	797	0.41%	822	0.48%	3380	0.4%
30–39 years	1966	1.0%	1979	0.97%	1791	0.92%	1587	0.92%	7323	1.0%
40–49 years	4349	2.2%	4868	2.38%	4373	2.25%	3857	2.23%	17,447	2.3%
50–54 years	5219	2.6%	6100	2.98%	5545	2.86%	5152	2.98%	22,016	2.9%
55–59 years	9648	4.9%	10,648	5.20%	9513	4.90%	8651	5.01%	38,460	5.0%
60–64 years	15,029	7.6%	15,768	7.70%	14,080	7.26%	11,922	6.90%	56,799	7.4%
65–74 years	45,785	23.1%	47,506	23.19%	41,019	21.14%	35,454	20.52%	169,764	22.0%
75–79 years	27,420	13.8%	30,314	14.80%	31,532	16.25%	30,704	17.77%	119,970	15.6%
80 + years	88,038	44.4%	86,840	42.39%	85,399	44.01%	74,600	43.18%	334,877	43.5%
**Deceased ICU patients receiving > 95 h ventilation**										
Case numbers	47,198		57,365		48,071		41,075		193,709	
**Gender**										
Male	30,095	63.8%	37,202	64.85%	30,989	64.47%	25,818	62.86%	124,104	64.1%
Female	17,089	36.2%	20,151	35.13%	17,076	35.52%	15,254	37.14%	69,570	35.9%
Diverse	1	0.0%	0	0.00%	0	0.00%	0	0.00%	1	0.0%
Unknown	13	0.0%	12	0.02%	6	0.01%	3	0.01%	34	0.0%
**Age groups**										
18–29 years	354	0.8%	345	0.60%	292	0.61%	285	0.69%	1276	0.7%
30–39 years	740	1.6%	814	1.42%	664	1.38%	550	1.34%	2768	1.4%
40–49 years	1610	3.4%	2046	3.57%	1572	3.27%	1317	3.21%	6545	3.4%
50–54 years	1876	4.0%	2483	4.33%	1976	4.11%	1704	4.15%	8039	4.2%
55–59 years	3377	7.2%	4216	7.35%	3290	6.84%	2913	7.09%	13,796	7.1%
60–64 years	5024	10.6%	6203	10.81%	4680	9.74%	3968	9.66%	19,875	10.3%
65–74 years	14,267	30.2%	17,369	30.28%	13,317	27.70%	10,946	26.65%	55,899	28.9%
75–79 years	7069	15.0%	9240	16.11%	8796	18.30%	7987	19.44%	33,092	17.1%
80 + years	12,881	27.3%	14,649	25.54%	13,484	28.05%	11,405	27.77%	52,419	27.1%

### Mortality

**Fig. 2:**
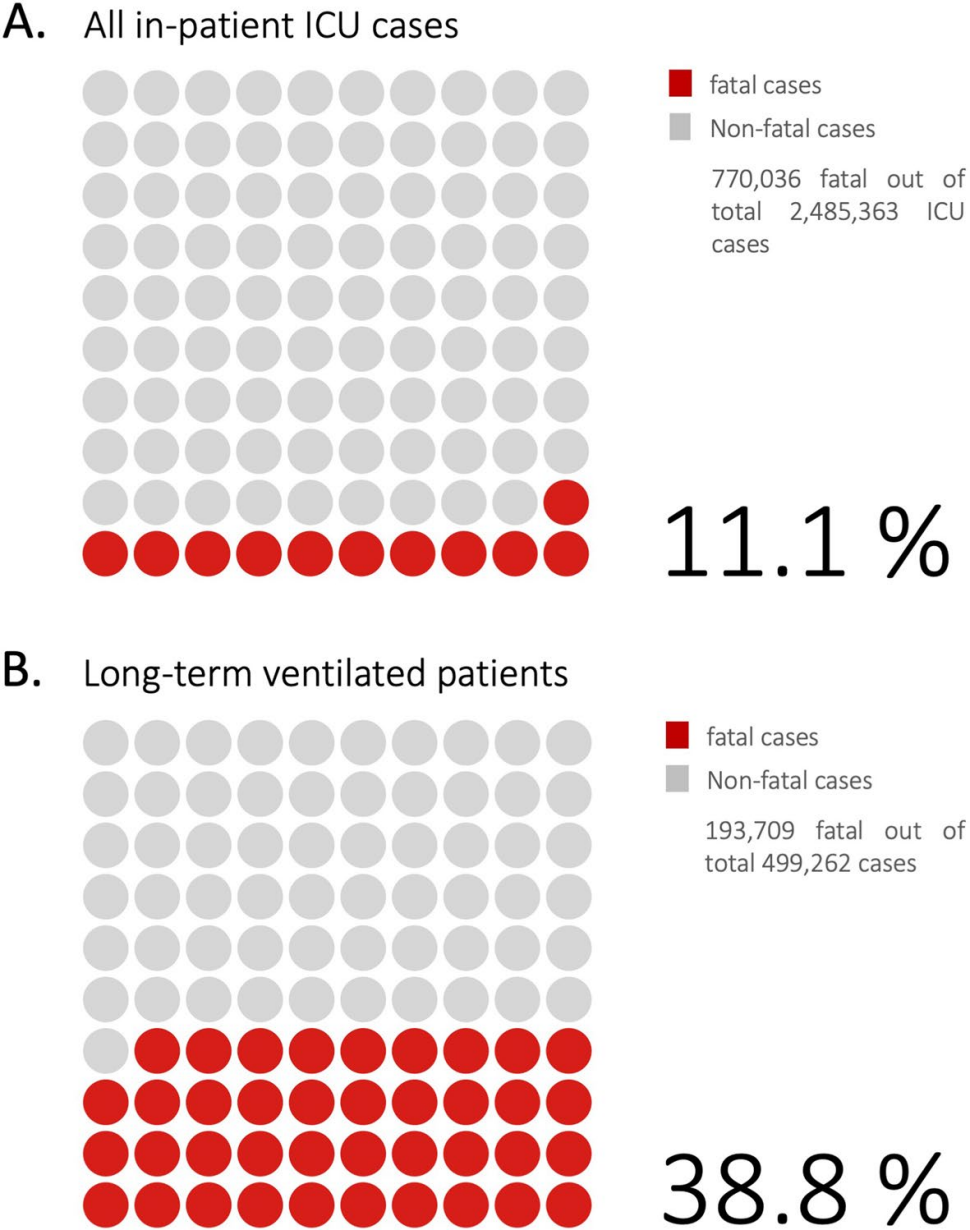
Mortality

**Fig. 3:**
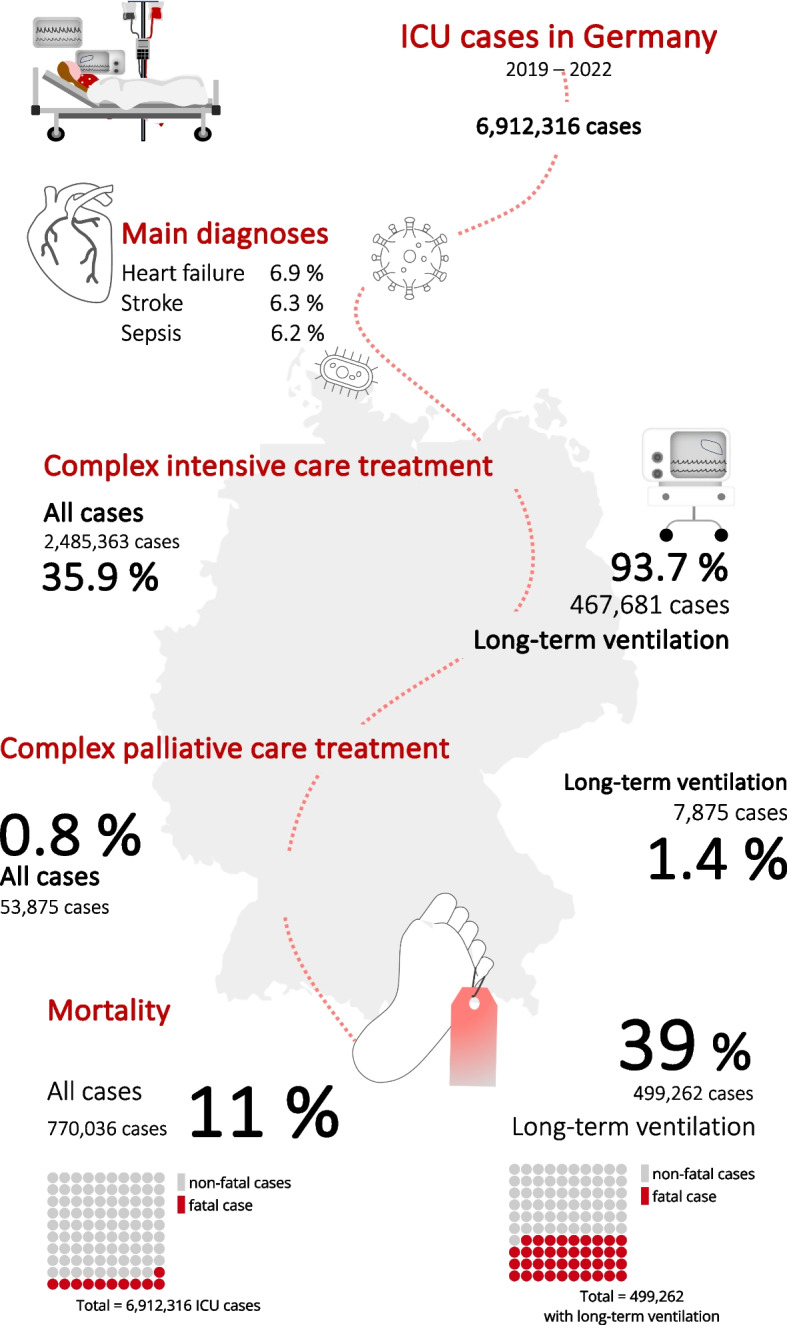
Infographic. Figure 3 summarizes the main results

770,036 ICU cases resulted in patient mortality, equating to a mortality rate of 11.1% based on the total number of ICU cases (Fig. 2). Among the deceased patients, 42.5% were female (327,556 cases), and 81.1% were ≥ 65 years old. For long-term ventilated patients, the mortality rate increased to 38.8% (193,709 cases). 73% of the long-term ventilated patients were ≥ 65 years (141,410 cases) and 64.1% male (124,104 cases). Women receiving intensive care and long-term ventilation had significantly lower mortality rates (entire ICU cohort: *p* < 0.001, Chi-square: 947.7; long-term ventilated cohort: *p* < 0.001, Chi-square: 11.9) (Fig. 3).

### Main diagnoses

Table 2 summarizes the primary diagnoses. Among all German ICU patients, the most common diagnoses were cerebral insult, acute myocardial infarction, and heart failure (9.5%, 6.1%, 3.5%, respectively). Of those diagnoses resulting in death in ICU cases heart failure, stroke, and sepsis were leading (6.9%, 6.3%, 6.2%, respectively). Viral pneumonia, chronic obstructive pulmonary disease, and acute myocardial infarction were the leading diagnoses among long-term ventilated patients (10%, 5.2%, 5.1%, respectively) as well as leading to fatal outcomes in this group (12.2%, 5.2%, 5.1%, respectively).

**Table 2:** Main diagnoses and selected diagnoses

		ICU patients ≥ 18		ICU patients ≥ 18, fatal cases		ventilation ≥ 95 h		ventilation > 95 h, fatal cases		ICU patients ≥ 18, receiving palliative care		ventilation > 95 h, receiving palliative care	
Total case numbers		6,912,316		770,036		499,262		193,709		57,116		7,501	
**ICD-10-GM code**	**Description**	**cases**	**%**	**cases**	**%**	**cases**	**%**	**cases**	**%**	**cases**	**%**	**cases**	**%**
C34	Lung cancer	70,386	1.0%	12,641	1.6%	4,123	0.8%	2,464	1.3%	4,212	7.4%	306	4.1%
I63	Stroke	662,466	9.6%	48,296	6.3%	17,965	3.6%	6,574	3.4%	3,350	5.9%	270	3.6%
I50	Heart failure	243,253	3.5%	53,489	6.9%	20,712	4.1%	9,320	4.8%	1,951	3.4%	163	2.2%
A40-A41	Streptococcal and other sepsis	138,790	2.0%	47,661	6.2%	24,934	5.0%	11,280	5.8%	1,737	3.0%	298	4.0%
C25	Pancreatic malignoma	28,727	0.4%	3,997	0.5%	1,568	0.3%	760	0.4%	1,466	2.6%	60	0.8%
J44		134,503	1.9%	20,920	2.7%	25,945	5.2%	6,696	3.5%	1,338	2.3%	272	3.6%
C79.3	Secondary malignant new formation of the brain and the meninges	19,242	0.3%	1,578	0.2%	638	0.1%	343	0.2%	1,206	2.1%	76	1.0%
C79.5	Secondary malignant new formation of the bone and the bone marrow	10,371	0.2%	1,460	0.2%	362	0.1%	226	0.1%	1,142	2.0%	45	0.6%
C56	Ovarian malignoma	16,068	0.2%	1,146	0.1%	573	0.1%	191	0.1%	956	1.7%	78	1.0%
S72	Fracture of the femur	198,651	2.9%	23,836	3.1%	4,959	1.0%	2,429	1.3%	903	1.6%	31	0.4%
C20	rectum malignoma	38,657	0.6%	2,481	0.3%	1,504	0.3%	619	0.3%	754	1.3%	65	0.9%
S06	Intracranial injury	137,738	2.0%	17,314	2.2%	14,759	3.0%	4,265	2.2%	684	1.2%	173	2.3%
I21	Acute myocardial infarction	401,331	5.8%	46,634	6.1%	25,327	5.1%	10,487	5.4%	668	1.2%	157	2.1%
J09-J12	Viral pneumonia	119,063	1.7%	41,720	5.4%	51,271	10.3%	23,725	12.2%	438	0.8%	204	2.7%
I35	Non-rheumatic aortic valve diseases	122,961	1.8%	5,715	0.7%	5,206	1.0%	1,810	0.9%	140	0.2%	20	0.3%
I48	Atrial fibrillation and atrial flutter	138,509	2.0%	3,545	0.5%	1,375	0.3%	618	0.3%	80	0.1%	0	0.0%
J80	Acute respiratory distress syndrome of children, adolescents and adults [ARDS]	12,371	0.2%	5,731	0.7%	9,399	1.9%	4,243	2.2%	78	0.1%	65	0.9%
I46	Cardiac arrest	17,272	0.2%	14,056	1.8%	3,989	0.8%	2,883	1.5%	76	0.1%	54	0.7%
I25	Chronic ischemic heart disease	110,755	1.6%	3,648	0.5%	4,712	0.9%	1,364	0.7%	56	0.1%	5	0.1%
G62	Other polyneuropathies / Critical illness polyneuropathy	10,950	0.2%	1,331	0.2%	7,148	1.4%	1,089	0.6%	47	0.1%	39	0.5%
G45	Cerebral transient ischemia and related syndromes	393,944	5.7%	1,364	0.2%	205	0.0%	44	0.0%	30	0.1%	0	0.0%
F10	Mental and behavioral disorders caused by alcohol	72,448	1.0%	313	0.0%	523	0.1%	105	0.1%	0	0.0%	0	0.0%
N17	Acute kidney failure	64,266	0.9%	12,841	1.7%	3,548	0.7%	1,706	0.9%	0	0.0%	0	0.0%
I69	Pneumonia caused by solid and liquid substances/vomit	27,583	0.4%	9,598	1.2%	5,845	1.2%	2,256	1.2%	0	0.0%	0	0.0%

### Treating hospitals

Hospitals with more than 1,000 beds treated 18.2% of all adult ICU cases (1,257,896 cases), primarily in publicly owned hospitals. These hospitals also treated 24.5% (122,171 cases) of long-term ventilated patients. Supplement Table 2 provides a detailed overview of bed capacities and hospital ownership.

### Palliative care treatment of ICU patients

53,875 of the 6,912,316 in-patient ICU cases (0.8%) in Germany received palliative care during the same hospital stay. Among these, 48.4% were female and 71% were aged ≥ 65 years. Female ICU patients received palliative care significantly more often than male ICU patients (OR: 1.18; 95% CI: 1.16 – 1.2; *p* < 0.0001, Table 3). Among the 56,328 palliative care treatments, 32.6% were classified as complex palliative care, 34.3% as extensive specialized palliative care, and 37.6% involved specialized palliative care provided by a consultation service (multiple coding possible).

**Table 3:** Palliative and intensive care treatments in hospital cases involving intensive care

		2022		2021		2020		2019			
**Case with patients receiving intensive and palliative care in the same hospital stay**											
Case numbers		14,473		13,904		13,935		11,563		53,875	
**Gender**											
Male		7383	51.0%	7211	51.9%	7211	52.0%	5972	51.6%	27,817	51.6%
Female		7087	49.0%	6693	48.1%	6693	48.0%	5590	48.3%	26,054	48.4%
Diverse		0	0.0%	0	0.0%	0	0.0%	0	0.0%	0	0.0%
Unknown		3	0.0%	0	0.0%	0	0.0%	1	0.0%	4	0.0%
**Age groups**											
18–29 years		102	0.7%	86	0.6%	86	0.6%	98	0.8%	371	0.7%
30–39 years		260	1.8%	247	1.8%	247	1.7%	224	1.9%	970	1.8%
40–49 years		511	3.5%	512	3.7%	512	3.8%	482	4.2%	2030	3.8%
50–54 years		622	4.3%	649	4.7%	649	4.7%	621	5.4%	2548	4.7%
55–59 years		1024	7.1%	1102	7.9%	1102	7.8%	922	8.0%	4134	7.7%
60–64 years		1507	10.4%	1452	10.4%	1452	10.5%	1133	9.8%	5558	10.3%
65–74 years		3746	25.9%	3530	25.4%	3530	24.8%	2845	24.6%	13,571	25.2%
75–79 years		1850	12.8%	1895	13.6%	1895	15.0%	1998	17.3%	7836	14.5%
80 + years		4851	33.5%	4431	31.9%	4431	31.1%	3240	28.0%	16,857	31.3%
**Total number of palliative care codes (ICD-10-GM: 8–982, 8-98e, 8-98 h)**										**56,328**	
**8–982**	**Palliative medical complex treatment**									**17,555**	31.2%
8–982.0	Palliative medical complex treatment: Up to 6 days of treatment	1219		1202		1047		1212		4680	8.3%
8–982.1	Palliative medical complex treatment: At least 7 to a maximum of 13 days of treatment	2138		2014		1743		1616		7511	13.3%
8–982.2	Palliative medical complex treatment: At least 14 to a maximum of 20 days of treatment	862		831		728		650		3071	5.5%
8–982.3	Palliative medical complex treatment: At least 21 days of treatment	686		609		513		485		2293	4.1%
**8-98e**	**Specialized inpatient palliative medical complex treatment on a palliative care unit**									**18,481**	32.8%
8-98e.0	Specialized inpatient palliative medical complex treatment: Up to 6 days of treatment	1321		1862		1873		1928		6984	12.4%
8-98e.1	Specialized inpatient palliative medical complex treatment: At least 7 to a maximum of 13 days of treatment	1213		1471		1640		1686		6010	10.7%
8-98e.2	Specialized inpatient palliative medical complex treatment: At least 14 to a maximum of 20 days of treatment	652		810		771		849		3082	5.5%
8-98e.3	Specialized inpatient palliative medical complex treatment: At least 21 days of treatment	551		621		612		621		2405	4.3%
**8-98 h**	**Specialized palliative medical complex treatment by a palliative care service**									**20,292**	36.0%
8-98 h.00	Specialized palliative medical complex treatment through a palliative care service: Through an internal palliative care service: Up to less than 2 h	579		923		1057		1059		3618	6.4%
8-98 h.01	Specialized palliative medical complex treatment through a palliative care service: Through an internal palliative care service: 2 to less than 4 h	1121		1353		1490		1629		5593	9.9%
8-98 h.02	Specialized palliative medical complex treatment through a palliative care service: Through an internal palliative care service: 4 to less than 6 h	730		1000		1048		1139		3917	7.0%
8-98 h.03	Specialized palliative medical complex treatment through a palliative care service: Through an internal palliative care service: 6 to less than 9 h	511		769		851		959		3090	5.5%
8-98 h.04	Specialized palliative medical complex treatment through a palliative care service: Through an internal palliative care service: 9 to less than 12 h	247		353		398		453		1451	2.6%
8-98 h.05	Specialized palliative medical complex treatment through a palliative care service: Through an internal palliative care service: 12 to less than 15 h	134		214		218		270		836	1.5%
8-98 h.06	Specialized palliative medical complex treatment through a palliative care service: Through an internal palliative care service: 15 to less than 20 h	126		181		224		222		753	1.3%
8-98 h.07	Specialized palliative medical complex treatment through a palliative care service: Through an internal palliative care service: 20 to less than 25 h	57		104		99		129		389	0.7%
8-98 h.08	Specialized palliative medical complex treatment through a palliative care service: Through an internal palliative care service: 20 to less than 25 h	47		79		76		101		303	0.5%
8-98 h.09	Specialized palliative medical complex treatment through a palliative care service: Through an internal palliative care service: 20 to less than 25 h	32		21		40		32		125	0.2%
8-98 h.0a	Specialized palliative medical complex treatment through a palliative care service: Through an internal palliative care service: 45 to less than 55 h			12		10		12		34	0.1%
8-98 h.0b	Specialized complex palliative care treatment through a palliative care service: Through an internal palliative care service: 55 or more hours	13		14		10		25		62	0.1%
8-98 h.10	Specialized palliative medical complex treatment by a palliative service: By an external palliative service: Up to less than 2 h			19		12		8		39	0.1%
8-98 h.11	Specialized palliative medical complex treatment by a palliative care service: By an external palliative care service: 2 to less than 4 h			24		12		14		50	0.1%
8-98 h.12	Specialized palliative medical complex treatment by a palliative care service: By an external palliative care service: 4 to less than 6 h	7		7						14	0.0%
8-98 h.13	Specialized palliative medical complex treatment by a palliative care service: By an external palliative care service: 6 to less than 9 h	5		6		7				18	0.0%
**Case with long-term ventilated patients > 95 h receiving palliative care in the same hospital stay**											
Case numbers		1,803		1,949		1,777		1,537		7,066	
**Gender**											
Male		1069	59.3%	1128	57.9%	1037	58.4%	899	58.5%	4133	58.5%
Female		734	40.7%	821	42.1%	740	41.6%	638	41.5%	2933	41.5%
Diverse		0	0.0%	0	0.0%	0	0.0%	0	0.0%	0	0.0%
Unknown		0	0.0%	0	0.0%	0	0.0%	0	0.0%	0	0.0%
**Age groups**											
18–29 years		27	1.5%	21	1.1%	12	0.7%	18	1.2%	78	1.1%
30–39 years		50	2.8%	43	2.2%	45	2.5%	48	3.1%	186	2.6%
40–49 years		69	3.8%	91	4.7%	67	3.8%	57	3.7%	284	4.0%
50–54 years		93	5.2%	103	5.3%	85	4.8%	98	6.4%	379	5.4%
55–59 years		152	8.4%	184	9.4%	146	8.2%	139	9.0%	621	8.8%
60–64 years		190	10.5%	237	12.2%	199	11.2%	152	9.9%	778	11.0%
65–74 years		569	31.6%	566	29.0%	518	29.2%	425	27.7%	2078	29.4%
75–79 years		222	12.3%	276	14.2%	275	15.5%	276	18.0%	1049	14.8%
80 + years		431	23.9%	428	22.0%	430	24.2%	324	21.1%	1613	22.8%
**Total number of palliative care codes (ICD-10-GM: 8–982, 8-98e, 8-98 h**										**7338**	
**8–982**	**Palliative medical complex treatment**									**1797**	24.5%
8–982.0	Palliative medical complex treatment: Up to 6 days of treatment	157		164		134		143		598	8.1%
8–982.1	Palliative medical complex treatment: At least 7 to a maximum of 13 days of treatment	211		175		165		142		693	9.4%
8–982.2	Palliative medical complex treatment: At least 14 to a maximum of 20 days of treatment	69		72		60		60		261	3.6%
8–982.3	Palliative medical complex treatment: At least 21 days of treatment	85		51		61		48		245	3.3%
**8-98e**	**Specialized inpatient palliative medical complex treatment on a palliative care unit**									**2025**	27.6%
8-98e.0	Specialized inpatient palliative medical complex treatment: Up to 6 days of treatment	194		266		273		272		1005	13.7%
8-98e.1	Specialized inpatient palliative medical complex treatment: At least 7 to a maximum of 13 days of treatment	105		115		162		125		507	6.9%
8-98e.2	Specialized inpatient palliative medical complex treatment: At least 14 to a maximum of 20 days of treatment	62		71		77		62		272	3.7%
8-98e.3	Specialized inpatient palliative medical complex treatment: At least 21 days of treatment	54		63		66		58		241	3.3%
**8-98 h**	**Specialized palliative medical complex treatment by a palliative care service**									**3516**	47.9%
8-98 h.00	Specialized palliative medical complex treatment through a palliative care service: Through an internal palliative care service: Up to less than 2 h	128		144		175		159		606	8.3%
8-98 h.01	Specialized palliative medical complex treatment through a palliative care service: Through an internal palliative care service: 2 to less than 4 h	206		232		233		242		913	12.4%
8-98 h.02	Specialized palliative medical complex treatment through a palliative care service: Through an internal palliative care service: 4 to less than 6 h	143		164		182		144		633	8.6%
8-98 h.03	Specialized palliative medical complex treatment through a palliative care service: Through an internal palliative care service: 6 to less than 9 h	97		114		157		134		502	6.8%
8-98 h.04	Specialized palliative medical complex treatment through a palliative care service: Through an internal palliative care service: 9 to less than 12 h	56		61		91		80		288	3.9%
8-98 h.05	Specialized palliative medical complex treatment through a palliative care service: Through an internal palliative care service: 12 to less than 15 h	34		46		54		52		186	2.5%
8-98 h.06	Specialized palliative medical complex treatment through a palliative care service: Through an internal palliative care service: 15 to less than 20 h	22		33		50		48		153	2.1%
8-98 h.07	Specialized palliative medical complex treatment through a palliative care service: Through an internal palliative care service: 20 to less than 25 h	8		18		30		40		96	1.3%
8-98 h.08	Specialized palliative medical complex treatment through a palliative care service: Through an internal palliative care service: 20 to less than 25 h	9		23		20		28		80	1.1%
8-98 h.09	Specialized palliative medical complex treatment through a palliative care service: Through an internal palliative care service: 20 to less than 25 h	5		6		12		9		32	0.4%
8-98 h.0a	Specialized palliative medical complex treatment through a palliative care service: Through an internal palliative care service: 45 to less than 55 h									0	0.0%
8-98 h.0b	Specialized complex palliative care treatment through a palliative care service: Through an internal palliative care service: 55 or more hours	6		6				10		22	0.3%
8-98 h.10	Specialized palliative medical complex treatment by a palliative service: By an external palliative service: Up to less than 2 h									0	0.0%
8-98 h.11	Specialized palliative medical complex treatment by a palliative care service: By an external palliative care service: 2 to less than 4 h			5						5	0.1%
8-98 h.12	Specialized palliative medical complex treatment by a palliative care service: By an external palliative care service: 4 to less than 6 h									0	0.0%
8-98 h.13	Specialized palliative medical complex treatment by a palliative care service: By an external palliative care service: 6 to less than 9 h									0	0.0%

Among the 7,066 long-term ventilated ICU patients who received palliative care, 67.1% were aged ≥ 65, and 41.5% were female. The likelihood of long-term ventilated female and elderly patients receiving palliative care was significantly higher compared to the general ICU population (female: OR: 1.3; 95% CI: 1.19 – 1.31; *p* < 0.001; aged ≥ 65: OR: 1.42; 95% CI: 1.4 – 1.44; ICU cohort: OR: 1.26; 95% CI: 1.2 – 1.33; *p* < 0.001). Among these cases, 25.4% involved complex palliative medicine, 28.7% specialized palliative medicine, and 49.8% involved palliative medicine consultation services (multiple coding possible, Table 3).

## Discussion

This study provides insights into the frequency of ICU-related hospital cases, mortality rates, and the utilization of specialized palliative care in ICUs over four years in Germany. Key findings include:

- Intensive care was required in 11.2% of all hospital cases, with long-term ventilation >95 hours needed in 0.8% of cases.
- The overall ICU mortality rate was 11.1%, rising to 38.8% for long-term ventilation cases, predominantly among patients aged ≥65 and males.
- Complex or specialized palliative care was provided in 53,875 ICU cases (0.8%) and 7,066 long-term ventilation cases (1.4%).

This study examined the frequency of intensive care treatments and long-term ventilation. A previous German study analyzed hospital remuneration data to assess end-of-life intensive therapy rates between 2007 and 2015 [16]. In 2015, 3.9% of all hospital cases involved intensive care, with 30.5% requiring mechanical ventilation and 16.2% needing ventilation for more than 95 h [16]. ICU cases in Germany more than doubled by 2019, a trend not solely driven by the SARS-CoV-2 pandemic, which led to peak ICU hospitalizations in 2020. Despite the overall rise in ICU cases, long-term ventilation cases declined between 2015 and 2019 but peaked again in 2021. The sharp increase in ICU cases from 2015 to 2019 may be partly due to changes in data reporting, complicating direct comparisons. Data from 2007 to 2015 only included patients with a standard length of stay, whereas our dataset covers all ICU patients, regardless of length of stay (short, standard, or extended). Many ICU patients, particularly those requiring long-term ventilation, often exceed the standard length of stay. As ICU hospital cases increased between 2015 and 2019, the mortality rate declined from 14.4% to 11.1% [16]. This rate is consistent with ICU mortality rates reported in other EU member states, the US, Scotland, and Australia/New Zealand, which range from 9 to 12% [23–26].

In this study, we determined the frequency of intensive care treatments and long-term ventilation. A previous German study also analyzed hospital remuneration data to determine end-of-life intensive therapy rates between 2007 and 2015 [16]. In 2015, 3.9% of all hospital cases involved intensive care, with 30.5% needing mechanical ventilation and 16.2% requiring it for > 95 h [16]. ICU cases in Germany more than doubled till 2019. This increase is not solely due to the SARS-CoV-2 pandemic, which peaked ICU hospitalizations in 2020. Despite the overall rise in ICU cases, long-term ventilation cases decreased from 2015 to 2019, but peaked in 2021. The sharp increase in ICU cases from 2015 to 2019 might be attributed to changes in data reporting, complicating data comparison. Data from 2007 until 2015 only pertains to patients with a standard length of stay, while our dataset includes all ICU patients (short / standard / long stay). Nonetheless, many ICU patients, especially those on long-term ventilation, require extended treatment beyond the standard length of stay. Between 2015 and 2019, as ICU hospital cases increased, the mortality rate decreased from 14.4% to 11.1% [16]. The latter mortality rate aligns with reported ICU mortalities in other EU member states, the US, Scotland, and Australia and New Zealand, which range from 9 to 12% [23–25, 27].

Achieving seamless integration of palliative care into intensive and emergency medicine remains challenging [9]. In Germany, the rate of ICU cases with patients receiving specialized palliative care averaged 0.8%, and among those on long-term ventilation, it was 1.4%. This frequency has hardly increased compared to the reported 0.7% in 2015 (5,084 out of 736,444 intensive care treatments). Our analysis excludes palliative care units (‘special facilities’, ‘besondere Einrichtungen’) that provide specialized palliative care but operate and receive funding outside the DRG system. Approximately 70 of the 350 palliative care units function as these so-called "special facilities." Unlike specialized palliative care provided in dedicated palliative care units, complex palliative care and consultation services are only captured within the DRG system. Further, patients could be discharged directly from ICU into Specialized Palliative Home Care (SAPV). Since the remuneration of outpatient services is not dealt with by the hospital remuneration system, these cases cannot be accounted for. Other countries and centers reported higher frequencies of palliative care integration into intensive care. A 2013 U.S. study from Columbia University Medical Center found that 88% of elderly ICU patients had potential palliative care needs, with a 6-month mortality rate of 40%. However, only 2.6% received palliative care consultations from a multiprofessional team [8]. Another U.S. study on ventilated patients with high mortality risk reported that 9.4% received palliative care consultations, with older age but not gender influencing the likelihood of receiving palliative care [28].

We found that younger patients (< 65 years) and male patients were less likely to receive palliative care on ICU. These findings however do not imply a causal relationship, as critical potential confounding factors could not be analyzed. Nonetheless, our findings are consistent with previous studies reporting similar associations. Previous studies also suggest age and gender differences in access to palliative care, with inconsistent findings. Some studies indicate that women are more likely to access hospice care than men, others show no effect or the opposite [29]. Women may have different symptoms, preferences, and communication patterns in end-of-life care [30]. Access to palliative care varies with age, some studies suggesting limited access for patients over 85 years [29–31].

A core goal of palliative medicine is to enhance the quality of life for patients with potentially life-threatening illnesses. In intensive care, palliative medicine extends beyond end-of-life care — it improves quality of life, reduces the length of ICU stays, and enhances communication and satisfaction among patients and their families [1]. Since our data cannot capture essential aspects of palliative care (eg. symptom burden, quality of life, stress experienced by ICU patients and their families) an accurate assessment of actual palliative care needs is not possible. Additionally, not all ICU patients require specialized palliative care. Part of our observation period includes the SARS-CoV-2 pandemic, which posed unique challenges for patients, families, and healthcare teams. International studies have highlighted barriers to integrating palliative care into ICUs during the pandemic [32–35]. The pandemic introduced significant challenges, leading to fewer patients receiving specialized palliative care in some regions and a reduced assessment of palliative care needs [33]. Additionally, provision of palliative care itself underwent radical changes. Visits from relatives were often impossible, and personal communication with families and within medical teams was severely limited [36–38]. While new strategies emerged, such web-based communication and platforms, these could only partially compensate for the essential personal interactions in palliative medicine [39]. Moreover, palliative care faced new medical challenges, including the management of SARS-CoV-2 patients requiring extracorporeal membrane oxygenation and experiencing severe symptoms such as weakness, fatigue, shortness of breath, and significant family distress [34].

### Limitations

We acknowledge several limitations of our current study:

1. Our data rely on billed hospital cases, which may not directly reflect the number of individual patients.
2. Data collected indicates the rate of ICU—and palliative care during the same hospital stay. No conclusion on temporal association between both treatments (parallel or sequentially) can be drawn. The "real rate" of specialized palliative medical care involvement in ICU patients is likely to be lower, since ICU physicians may provide basic palliative medical treatment themselves.
3. Data spans the duration of the SARS-CoV-2 pandemic in Germany, potentially influencing diagnoses and treatment outcomes. However, data from 2019 (pre-Covid pandemic) is consistent with data from the following pandemic years 2020–2022.

## Conclusion

From 2019 to 2022, 11% of the 61.6 million adult hospital cases in Germany required ICU care, and 7% of these ICU cases (499,262 patients) needed long-term ventilation for over 95 h. Despite the severity of their conditions and high mortality rates, only 0.8% of ICU patients and 1.4% of those on long-term ventilation received specialized palliative care. These real-world data reveal the current level of palliative care integration in ICU treatment. There is a need to improve access to palliative care for ICU patients and their families with complex needs. Implementing a benchmarking process could help achieve this goal.

## Supplementary Information

Supplementary Material 1: Supplemental Table 1. Definition of OPS-Codes.

Supplementary Material 2: Supplemental Table 2. Hospital. The table presents a breakdown of the number of case treatments categorized by the ownership and bed capacity of the treating hospitals.

Supplementary Material 3: SupplementalTable 3. STROBE checklist.

Supplementary Material 4: Supplemental Fig. 1. OPS Code 8–980. Intensive care complex treatment (basic procedure), Case distribution of all ICU cases / long term ventilation and fatal cases (sum of complexity scores SAPS II and TISS, definition see Supplement Table 1).

Supplementary Material 5: Supplemental Fig. 2. OPS Code 8-98f. Specialized intensive medical treatment (basic procedure), Case distribution of all ICU cases / long term ventilation and fatal cases (sum of complexity scores SAPS II and TISS, definition see Supplement Table 1).

## Data Availability

The data is available upon request.

## Abbreviations

CI: Confidence Interval
DIVI: *Deutsche Interdisziplinäre Vereinigung für Intensiv- und Notfallmedizin* (German Society for Intensive Care Medicine and Emergency Care Medicine)
G-DRG: German Diagnosis Related Groups
ICD—10: International Statistical Classification of Diseases and Related Health Problems
ICU: Intensive Care Unit
InEK: *Institut für das Entgeltsystem im Krankenhaus* (Hospital Renumeration Institute)
OPS: *Operationen – und Prozeduren Schlüssel* (Operations and Procedural Catalogue)
OR: Odds Ratio
SAPV: Spezialisierte Ambulante Palliativversorgung (Specialized Palliative Home Care)
SICSAG: Scotland and Scottish Intensive Care Society Audit Group
STROBE: Strengthening the Reporting of Observational Studies in Epidemiology
