# Supplementary material for: Analyzing the use of specialized palliative care in intensive care unit patients in Germany: a cross-sectional study
Source: BMC Palliat Care. 2025 Mar 20;24:74. doi: 10.1186/s12904-025-01718-1 (PMC11924865; doi:10.1186/s12904-025-01718-1)
Supplement: Supplementary file 1 — Supplementary Material 1: Supplemental Table 1. Definition of OPS-Codes. [file 12904_2025_1718_MOESM1_ESM.docx]

Supplemental table 4:

Definition of OPS-Codes

**8-982 Complex palliative care**

*Not included:*

- Specialized inpatient complex palliative care treatment (8-98e)
- Specialized complex palliative care treatment by a palliative care service (8-98h)

*Required structural features:*

- Treatment led by a specialist with additional qualification in palliative medicine

*Minimum features:*

- Conducting a standardized palliative medicine basic assessment (PBA) at the start of treatment
- Holistic treatment for symptom control and psychosocial stabilization of patients with a progressive, advanced illness and limited life expectancy, if necessary, with the involvement of their relatives
- Creation and documentation of an individual treatment plan upon admission
- Patient-specific documentation of palliative medicine treatment goals and treatment results
- Weekly multi-professional team meeting with the presence of the medical treatment manager and at least one member of the nursing team and at least one other representative of the professional groups involved in patient care per full week
- Use of at least 2 of the following therapy areas: social work/social education, psychology, special education, physiotherapy/occupational therapy, artistic therapy (art and/or music therapy), relaxation therapy and conducting patient, relative and/or family discussions with a total of at least 6 hours per patient and full week patient-related in different combinations (the patient, relative and/or family discussions can be attended by all professional groups of the treatment team.) If two or more representatives of different professional groups of the treatment team are used simultaneously, the respective employee minutes are added up

8 8-982.0 Up to 6 days of treatment

8-982.1 At least 7 to a maximum of 13 days of treatment

8-982.2 At least 14 to a maximum of 20 days of treatment

8-982.3 At least 21 days of treatment

**8-98e Specialized inpatient palliative medical complex treatment** *Not included:*

- Complex palliative medical treatment (8-982)
- Specialized complex palliative medical treatment by a palliative care service (8-98h)

*Required structural features:*

- Existence of an independent palliative care unit (at least 5 beds) with a multi-professional team specializing in particularly complex and demanding palliative care
- Professional treatment management by a specialist with additional qualifications in palliative medicine and at least 6 months of experience in treating palliative patients in a palliative care ward or in another specialized palliative care facility. 24-hour professional treatment management can be ensured by on-call
- At least 7 hours of medical presence in the palliative care unit on working days
- Nursing management with proof of a recognized curricular additional qualification in palliative care of at least 160 hours and with at least 6 months of experience in a specialized palliative care facility
- Existence of specialized device-based palliative medical treatment procedures with the possibility of continuous monitoring, e.g., pain pumps and other continuous parenteral therapies for symptom control

*Minimum features:*

- Conducting a standardized palliative medicine basic assessment (PBA) at the start of treatment
- Daily multi-professional case discussion with attendance documentation
- Creation and documentation of an individual treatment plan upon admission
- Patient-specific documentation of palliative medicine treatment goals and treatment results
- Holistic treatment for symptom control and psychosocial stabilization of patients with a progressive, advanced illness and limited life expectancy, if necessary, with the involvement of their relatives
- Weekly multi-professional team meeting with the presence of the medical treatment manager and at least one member of the nursing team and at least one other representative of the professional groups involved in patient care per full week
- Use of at least 2 of the following therapy areas: social work/social education, special education, psychology, physiotherapy/occupational therapy, artistic therapy (art and/or music therapy), relaxation therapy and conducting patient, relative and/or family discussions with a total of at least 6 hours per patient and full week, patient-related in different combinations (The Patient, relative and/or family discussions can be carried out by all professional groups of the treatment team.) If two or more representatives of different professional groups of the treatment team are deployed simultaneously, the respective employee minutes are added up
- If necessary, referral to qualified and continuous support offers for relatives (also after the death of the patient)
- Referral and transfer to subsequent forms of general and specialized palliative care with particular consideration of emergency planning, structured instructions for relatives, social legal advice and referral, if necessary

8-98e.0 Up to 6 days of treatment

8-98e.1 At least 7 to a maximum of 13 days of treatment

8-98e.2 At least 14 to a maximum of 20 days of treatment

8-98e.3 At least 21 days of treatment

**8-98h Specialized complex palliative care treatment by a palliative care service**

*Not included:*

- Complex palliative care treatment (8-982)
- Specialized inpatient complex palliative care treatment (8-98e ff.)

A code from this area must only be entered once per in-patient stay

*Required structural features:*

- A cross-departmental, organizationally independent, multi-professional team specializing in complex palliative care (palliative care service), consisting of a medical service, a nursing service and at least one representative from another area: social work/social education, psychology/psychotherapy, physiotherapy, occupational therapy. It offers its services for the co-treatment of patients in a case-leading department and coordinates these with the case-leading department
- Medical treatment management by a specialist with additional qualification in palliative medicine and nursing management by a nursing professional with proof of a recognized curricular palliative care additional qualification of at least 160 hours (each with at least 6 months experience in specialized palliative care)
- 24-hour availability and, if professionally necessary, the presence of a specialist with at least 6 months experience in specialized palliative care who is familiar with the patients' current problems. Outside of regular working hours, this specialist does not have to be part of the organizationally independent palliative care service team, but must be familiar with the patients' current problems

*Minimum features:*

- Implementation of a standardized palliative medicine basic assessment (PBA) at the start of treatment by the palliative care service
- Creation of an individual treatment plan agreed with the case-leading department at the start of treatment by the palliative care service
- Patient-specific documentation of palliative medicine treatment goals and treatment results by the palliative care service
- Active, holistic treatment for symptom control and psychosocial stabilization of patients with a progressive, advanced illness and limited life expectancy, if necessary, with the involvement of their relatives, in addition to the treatment by the case-leading department
- Weekly team meeting of the palliative care service with the presence of the medical treatment manager and at least one member of the palliative care service nursing staff as well as at least one other representative of the professional groups of the palliative care service involved in patient care per full week
- Forward care planning and coordination of palliative care, e.g., by setting indications for mediation and transfer to subsequent forms of care in general and specialized palliative care with particular consideration of emergency advance planning, if necessary
- If necessary, referral to qualified and continuous support services for relatives
- The time spent by doctors from the palliative care service, nursing staff from the palliative care service and representatives of the above-mentioned areas of the palliative care service on the patient and on the patient's relatives/caregivers is added up over the entire inpatient stay and coded accordingly. If two or more representatives from different professional groups of the treatment team are deployed simultaneously, the respective employee minutes are added up

8-98h.0 By an internal palliative care service

Note: A code from this area is only to be used if the palliative care service of the hospital in which the patient is being treated as an inpatient is providing the palliative care

8-98h.00 Up to 2 hours

8-98h.01 2 to 4 hours

8-98h.02 4 to 6 hours

8-98h.03 6 to 9 hours

8-98h.04 9 to 12 hours

8-98h.05 12 to 15 hours

8-98h.06 15 to 20 hours

8-98h.07 20 to 25 hours

8-98h.08 25 to 35 hours

8-98h.09 35 to 45 hours

8-98h.0a 45 to 55 hours

8-98h.0b 55 or more hours

8-98h.1 By an external palliative care service

Note: A code from this area is only to be used if the palliative care service of an external service provider carries out the palliative medical treatment

8-98h.10 Up to 2 hours

8-98h.11 2 to 4 hours

8-98h.12 4 to 6 hours

8-98h.13 6 to 9 hours

8-98h.14 9 to 12 hours

8-98h.15 12 to 15 hours

8-98h.16 15 to 20 hours

8-98h.17 20 to 25 hours

8-98h.18 25 to 35 hours

8-98h.19 35 to 45 Hours

8-98h.1a 45 to less than 55 hours

8-98h.1b 55 or more hours

**8-980 Intensive care complex treatment (basic procedure)**

*Not included:*

- Intensive care monitoring without acute treatment of vital organ systems or short-term (< 24 hours) intensive care
- Short-term (< 24 hours) stabilization of patients after surgical interventions

*Required structural features:*

- Treatment led by a specialist with additional qualifications in intensive care medicine
- Team of nursing staff and doctors on call for acute treatment
- A constant medical presence in the intensive care unit must be guaranteed. The intensive care unit doctor can be called in for a short-term emergency within the hospital (e.g., resuscitation)

*Minimum features:*

- Continuous, 24-hour monitoring

The number of effort points is calculated from the sum of the daily SAPS II (without Glasgow Coma Scale) over the length of stay in the intensive care unit (total SAPS II) plus the sum of 10 daily complex services from the TISS catalogue over the length of stay in the intensive care unit. Special intensive care procedures, such as transfusion of plasma and plasma components, plasmapheresis and immunoadsorption, installation and operation of an ECMO/ECLS, measures as part of resuscitation, etc., must be coded separately. These codes must be given for patients who are 14 years or older when admitted to hospital.

8-980.0 1 to 184 effort points

8-980.1 185 to 552 effort points

8-980.10 185 to 368 effort points

8-980.11 369 to 552 effort points

8-980.2 553 to 1,104 effort points

8-980.20 553 to 828 effort points

8-980.21 829 to 1,104 effort points

8-980.3 1,105 to 1,656 effort points

8-980.30 1,105 to 1,380 effort points

8-980.31 1,381 to 1,656 effort points

8-980.4 1,657 to 2,208 effort points

8-980.40 1,657 to 1,932 effort points

8-980.41 1,933 to 2,208 effort points

8-980.5 2,209 to 2,760 effort points

8-980.50 2,209 to 2,484 effort points

8-980.51 2,485 to 2,760 effort points

8-980.6 2,761 to 3,680 effort points

8-980.60 2,761 to 3,220 effort points

8-980.61 3,221 to 3,680 effort points

8-980.7 3,681 to 4,600 effort points

8-980.8 4,601 to 5,520 effort points

8-980.9 5,521 to 7,360 effort points

8-980.a 7,361 to 9,200 effort points

8-980.b 9,201 to 11,040 effort points

8-980.c 11,041 to 13,800 effort points

8-980.d 13,801 to 16,560 effort points

8-980.e 16,561 to 19,320 effort points

8-980.f 19,321 or more effort points

**8-98f Specialized intensive medical treatment (basic procedure)**

*Not included:*

- Intensive care monitoring without acute treatment of vital organ systems or short-term (< 24 hours) intensive care
- Short-term (< 24 hours) stabilization of patients after surgical interventions

*Required structural features:*

- Treatment management by a specialist with additional qualification in intensive care medicine who carries out the majority of his medical work in the intensive care unit
- Team of nursing staff and doctors on call for acute treatment
- A specialist with additional qualification in intensive care medicine (the treatment manager or another specialist with additional qualification in intensive care medicine) must be present in the intensive care unit for at least 7 hours on weekdays between 6 a.m. and 10 p.m. Outside of this attendance time, a specialist with additional qualification in intensive care medicine must be available to the patient within 30 minutes
- Constant medical presence in the intensive care unit must be guaranteed. The intensive care unit doctor can be called in for a short-term emergency within the hospital (e.g., resuscitation)
- 24-hour availability of the following procedures at the hospital location:
- Mechanical ventilation
- Non-invasive and invasive monitoring
- Continuous and intermittent renal replacement procedures
- Endoscopy of the gastrointestinal tract and the tracheobronchial system
- Intracranial pressure measurement or hybrid operating room for cardiovascular interventions
- Transoesophageal echocardiography
- 24-hour availability of 3 of the following 4 procedures at the hospital location:
- Radiological diagnostics using CT and MRI
- Interventional cardiology with acute PTCA
- Interventional (neuro)radiology with acute endovascular therapy of vascular and organ injuries and/or cerebral vascular occlusions
- Laboratory services (e.g., blood gas analyses, determination of electrolytes, lactate, differential blood count, coagulation, retention values, Enzymes, inflammation parameters, including procalcitonin, toxic screen). Specialized laboratory diagnostics may also be carried out in external laboratories
- At least 6 of the following 8 specialist areas are available within a maximum of 30 minutes at the hospital location as clinical consultation services (belonging to the hospital or from neighbouring hospitals): cardiology, gastroenterology, neurology, anaesthesiology, visceral surgery, trauma surgery, vascular surgery, neurosurgery
- Daily availability (including weekends) of physiotherapy services

*Minimum features:*

- Continuous, 24-hour monitoring
- A specialist with additional qualification in intensive care medicine (the treatment manager or another specialist with additional qualification in intensive care medicine) must carry out at least one round daily

The number of effort points is calculated from the sum of the daily SAPS II (without Glasgow Coma Scale) over the length of stay in the intensive care unit (total SAPS II) plus the sum of 10 daily complex services from the TISS catalogue over the length of stay in the intensive care unit. Special intensive care procedures, such as transfusion of plasma and plasma components, plasmapheresis and immunoadsorption, installation and operation of an ECMO/ECLS, measures as part of resuscitation, etc., must be coded separately. These codes must be given for patients who are 14 years or older when admitted to hospital

8-98f.0 1 to 184 effort points

8-98f.1 185 to 552 effort points

8-98f.10 185 to 368 effort points

8-98f.11 369 to 552 effort points

8-98f.2 553 to 1,104 effort points

8-98f.20 553 to 828 effort points

8-98f.21 829 to 1,104 effort points

8-98f.3 1,105 to 1,656 effort points

8-98f.30 1,105 to 1,380 effort points

8-98f.31 1,381 to 1,656 effort points

8-98f.4 1,657 to 2,208 effort points

8-98f.40 1,657 to 1,932 effort points

8-98f.41 1,933 to 2,208 effort points

8-98f.5 2,209 to 2,760 effort points

8-98f.50 2,209 to 2,484 effort points

8-98f.51 2,485 to 2,760 effort points

8-98f.6 2,761 to 3,680 effort points

8-98f.60 2,761 to 3,220 effort points

8-98f.61 3,221 to 3,680 effort points

8-98f.7 3,681 to 4,600 effort points

8-98f.8 4,601 to 5,520 effort points

8-98f.9 5,521 to 7,360 effort points

8-98f.a 7,361 to 9,200 effort points

8-98f.b 9,201 to 11,040 effort points

8-98f.c 11,041 to 13,800 effort points

8-98f.d 13,801 to 16,560 effort points

8-98f.e 16,561 to 19,320 effort points

8-98f.f 19,321 or more effort points
