# Supplementary material for: Analyzing the use of specialized palliative care in intensive care unit patients in Germany: a cross-sectional study
Source: BMC Palliat Care. 2025 Mar 20;24:74. doi: 10.1186/s12904-025-01718-1 (PMC11924865; doi:10.1186/s12904-025-01718-1)
Supplement: Supplementary file 3 — Supplementary Material 3: SupplementalTable 3. STROBE checklist. [file 12904_2025_1718_MOESM3_ESM.docx]

STROBE Statement—checklist of items that should be included in reports of observational studies

|  | Item No. | Recommendation | Page  No. | Relevant text from manuscript |
| --- | --- | --- | --- | --- |
| **Title and abstract** | 1 | (*a*) Indicate the study’s design with a commonly used term in the title or the abstract | 1 | Analysing the use of specialized palliative care in intensive care unit patients in Germany: cross-sectional study spanning all ICU cases in Germany over four years |
|  |  | (*b*) Provide in the abstract an informative and balanced summary of what was done and what was found | 3 | *Methods*  Billing data (2019 -2022) from the InEK was used in this cross-sectional study on all billed adult ICU cases. Data included case numbers, demographics, diagnoses, treatment procedures, ventilation (≥95h), palliative care frequency.  *Results*  61,591,299 adult cases were treated, 11.2% (6,912,316) requiring ICU and 499,262 (7.2%) needing long-term ventilation. 44.2% of all ICU cases and 36.2% of long-term ventilated patients were female (p < 0.0001). ICU mortality was 11.1%, long-term ventilation mortality was 38.8%; higher in men and patients aged ≥ 65 (p < 0.001). Leading diagnoses for ICU deaths: heart failure (6.9%), stroke (6.3%), sepsis (6.2%). 0.8% of ICU cases and 1.4% of long-term ventilated cases received specialized palliative care, with a higher proportion of females (p < 0.0001). Most palliative care patients were aged ≥ 65. |
| Introduction | | | |  |
| Background/rationale | 2 | Explain the scientific background and rationale for the investigation being reported | 4 and 5 | … palliative care principles, including thorough symptom management and clear communication, have become integral components of corresponding intensive care recommendations (Truog et al., 2008; Michels et al., 2023b). Moreover, various medical society and guidelines recommend an early integration of palliative care for seriously ill and ICU patients (Truog *et al.*, 2008; Ferrell *et al.*, 2017; De Simone *et al.*, 2024). … Reliable international and German data on the integration of palliative and intensive care medicine are limited. |
| Objectives | 3 | State specific objectives, including any prespecified hypotheses | 5 | The aim of the present study was to determine the frequency of specialized palliative care treatment in hospitalized patients admitted to German ICUs. |
| Methods | | | |  |
| Study design | 4 | Present key elements of study design early in the paper | 1,3 and 6 | cross sectional study |
| Setting | 5 | Describe the setting, locations, and relevant dates, including periods of recruitment, exposure, follow-up, and data collection | 2 and 5 | .. period from 01.01.2019 to 31.12.2022 … relied on public data … It is compulsory for all German Hospitals to send their data … to InEK GmbH, … (to) aggregate received data making it publicly available via the InEK Browser (§21 Hospital Fees Act). |
| Participants | 6 | (*a*) *Cohort study*—Give the eligibility criteria, and the sources and methods of selection of participants. Describe methods of follow-up  *Case-control study*—Give the eligibility criteria, and the sources and methods of case ascertainment and control selection. Give the rationale for the choice of cases and controls  *Cross-sectional study*—Give the eligibility criteria, and the sources and methods of selection of participants | 5 | Data for all billed hospital cases involving ICU care meeting the following criteria was obtained:  1. > 18 years of age  2. total number of hospital cases,  3. total number involving ICU care  4. all cases on ICU requiring > 95h ventilation to exclude ICU patients with non-life threating illnesses |
|  |  | (*b*) *Cohort study*—For matched studies, give matching criteria and number of exposed and unexposed  *Case-control study*—For matched studies, give matching criteria and the number of controls per case |  |  |
| Variables | 7 | Clearly define all outcomes, exposures, predictors, potential confounders, and effect modifiers. Give diagnostic criteria, if applicable | 6-7 suppl | Data extracted included:  1.case numbers for each cohort  2.demographics (age groups as predefined by InEK browser, sex distribution classified into female, male, diverse, unknown)  3.case distribution among hospitals according to bed capacity and ownership  4.count of primary diagnoses and treatment procedures  5. Administration of specialized palliative care according to Operations and Procedure Codes (OPS 8-982, 8-98e, 8-98h) and complex intensive care treatment (OPS 8-980, 8-98f, for code definition see suppl. table 2).  Long-term ventilation was defined according to the classification of the German Diagnosis Related Groups (G-DRG) system as ventilation lasting longer than 95 hours in conjunction with intensive care complex treatment (Institut für das Entgeltsystem im Gesundheitsweisen GmbH, 2024)  Supplement with definition of OPS codes |
| Data sources/ measurement | 8* | For each variable of interest, give sources of data and details of methods of assessment (measurement). Describe comparability of assessment methods if there is more than one group | *6* | InEK Browser, aggregated Hospital billing data from all German Hospitals. Results structured by variables defined above |
| Bias | 9 | Describe any efforts to address potential sources of bias | 7 | Bias  We minimized selection bias by including all consecutive adult ICU admissions in Germany from 2019 to 2022, ensuring a comprehensive and representative sample. However, our analysis pertains to hospital cases rather than individual patients. Reported data is complete considering all billed cases reported to and made available by the InEK from all German Hospitals with the exception of potential results of less than four cases for data protection. Considering the high volume of cases analysed, this small number of cases should not affect the overall outcome. To reduce measurement bias, we extracted data on palliative care consultations and intensive care treatments using predefined OPS and ICD-10 codes, ensuring reliable and consistent identification of in-patient ICU cases receiving specialized palliative care. While OPS and ICD-10 classifications are well established, we cannot entirely rule out misclassifications. Measurement errors are minimal in our study based on billing data. The study aimed to determine the frequency of palliative medical treatment during ICU stays and long-term ventilation, using descriptive statistics, which minimizes the impact of confounding bias. However, information on key demographic and clinical variables, such as age, gender, and severity of illness, are given in detail. |
| Study size | 10 | Explain how the study size was arrived at | 5 - 6 | Study size is a function of the output given by the InEK Browser: all billed cases spanning the requested time frame are being displayed. |

Continued on next page

| Quantitative variables | 11 | Explain how quantitative variables were handled in the analyses. If applicable, describe which groupings were chosen and why | 7 | Data were obtained from the InEK data browser and organized using Microsoft Excel for Mac (version 16.78, Microsoft Corporation, Redmond, Washington, USA). Statistical analyses and graphical representations were conducted using GraphPad Prism 9 for macOS (version 9.5.0, GraphPad Software, Inc., La Jolla, USA).  Groupings were predefined by the InEK Browser (age and gender). Diagnoses are coded using the DRG System. |
| --- | --- | --- | --- | --- |
| Statistical methods | 12 | (*a*) Describe all statistical methods, including those used to control for confounding | 7 and 8 | -Descriptive statistics were utilized to calculate the percentages of patients who passed away and those who received treatment.  -The Chi-square test with Yates' correction was employed to examine differences in sex distributions across various subgroups  -A two-sided significance level of α = 0.05 was applied, with a Bonferroni correction for multiple comparisons (n = 8) Thus, the adjusted significance level was set at 0.006. To assess the association between two categorical variables in 2 x 2 contingency tables, we calculated odds ratios (OR) along with their corresponding 95 % confidence intervals (95 %-CI) |
|  |  | (*b*) Describe any methods used to examine subgroups and interactions | 7 | To assess the association between two categorical variables in 2 x 2 contingency tables, we calculated odds ratios (OR) along with their corresponding 95 % confidence intervals (95 %-CI) |
|  |  | (*c*) Explain how missing data were addressed | 6 | Reported data is complete considering all billed cases reported to and made available by the InEK from all German Hospitals with the exception of potential results of less than four cases for data protection. Considering the high volume of cases analysed, this small number of cases should not affect the overall outcome. |
|  |  | (*d*) *Cohort study*—If applicable, explain how loss to follow-up was addressed  *Case-control study*—If applicable, explain how matching of cases and controls was addressed  *Cross-sectional study*—If applicable, describe analytical methods taking account of sampling strategy |  | n/a |
|  |  | (*e*) Describe any sensitivity analyses |  | n/a |
| Results | | | | |
| Participants | 13* | (a) Report numbers of individuals at each stage of study—e.g., numbers potentially eligible, examined for eligibility, confirmed eligible, included in the study, completing follow-up, and analysed | Table 2 & Figure 2, p 7 & Supplement | … 61,591,299 adult hospital cases. … intensive care in 6,912,316 hospital cases … and ventilation > 95 hours in 499,262 …A complex or specialized ICU therapy (OPS codes 8-980 or 8-98f) was conducted and billed in 2,485,363 ICU cases …and in 467,681 ICU cases with long-term ventilation… |
|  |  | (b) Give reasons for non-participation at each stage | P 6 & 10 | If case numbers were < 4, they would not be displayed for privacy reasons. Around 70 palliative care units in Germany are classified as "special facilities" (*besondere Einrichtungen*), meeting specific criteria and remunerating outside the DRG system. These cannot be accounted for in our dataset. Theoretically, patients could be discharged directly from ICU into Specialized Palliative Home Care (SAPV). Since the remuneration of outpatient services is not dealt with by the hospital remuneration system, these cases cannot be accounted for. |
|  |  | (c) Consider use of a flow diagram | infographic | infographic |
| Descriptive data | 14* | (a) Give characteristics of study participants (e.g., demographic, clinical, social) and information on exposures and potential confounders | 9 | Age:  26.7 % of all hospital patients were ≥65 (32,252,121 cases), whilst 63.3% of ICU patients and 61.8% of long-term ventilated patients (p < 0.0001) were ≥65  Gender:  Female 52.7 % of all hospital cases (32,449,759 cases), 44.2 % of ICU cases (3,054,198 patients) and 36.2 % of long-term ventilation cases (180,799 cases; p < 0.0001).  *Mortality:* 770,036 ICU Patients died (11.1 %); 42.5 % were female (327,556 cases), and 81.1 % were ≥65 years old; in long-term ventilated patients: 38.8 % (193,709 patients); of these 73 % were ≥ 65 years (141,410 cases) and 64.1 % male (124,104 cases). Women receiving intensive care and long-term ventilation had significantly lower mortality rates (entire ICU cohort: p < 0.001, Chi-square: 947.7; long-term ventilated cohort: p < 0.001, Chi-square: 11.9).  *Main diagnosis*  five leading main diagnoses for ICU patients:  -cerebral insult (9.5 %),  -acute myocardial infarction (6.1 %),  -heart failure (3.5 %),  sepsis (2 %),  -viral pneumonia (1.9 %). pneumonia (12.2 %), chronic obstructive pulmonary disease (5.2 %) and acute myocardial infarction (5.1 %) the most common primary diagnoses in fatal ICU cases with long-term ventilated patients. |
|  |  | (b) Indicate number of participants with missing data for each variable of interest |  | n/a |
|  |  | (c) *Cohort study*—Summarise follow-up time (e.g., average and total amount) |  | n/a |
| Outcome data | 15* | *Cohort study*—Report numbers of outcome events or summary measures over time |  | *n/a* |
|  |  | *Case-control study—*Report numbers in each exposure category, or summary measures of exposure |  | *n/a* |
|  |  | *Cross-sectional study—*Report numbers of outcome events or summary measures | *Page 10* | *Palliative care treatment of ICU patients*  53,875 of the 6,912,316 in-patient ICU cases (0.8 %) in Germany received palliative care during the same hospital stay. …48.4 % were female and 71 % were aged ≥ 65 years.  Among the 7,066 long-term ventilated ICU patients who received palliative care, 67.1 % were aged ≥ 65, and 41.5 % were female.  Viral pneumonia … most prevalent primary diagnosis among long-term ventilated patients (10 %), followed by chronic obstructive pulmonary disease (5.2 %), acute myocardial infarction (5.1 %), and sepsis (5 %). Heart failure (6.9 %), stroke (6.3 %) and sepsis (6.2 %) were the most common primary diagnoses in all ICU hospital cases resulting patient’s death. |
| Main results | 16 | (*a*) Give unadjusted estimates and, if applicable, confounder-adjusted estimates and their precision (e.g., 95% confidence interval). Make clear which confounders were adjusted for and why they were included | Page 10 | Female ICU patients were significantly more likely to receive palliative care compared to those without palliative care (OR: 1.18; 95 % CI: 1.16 – 1.2; p < 0.0001). … Palliative care physicians provided complex palliative care treatment in 17,555 out of 53,875 cases (32.6 %). Additionally, more extensive specialized palliative care treatment was administered in 18,481 cases (34.3 %) and additional 20,292 cases (37.6 %) involved specialized palliative care by a palliative care consultation service (multiple coding possible) …  … likelihood of long-term ventilated female and elderly patients receiving palliative care was significantly higher compared to the general ICU population (female: OR: 1.3; 95 % CI: 1.19 – 1.31; p < 0.001; aged ≥ 65: OR: 1.42; 95 % CI: 1.4 – 1.44; ICU cohort: OR: 1.26; 95 % CI: 1.2 – 1.33; p < 0.001). Among these cases, 1,797 involved complex palliative medicine (25.4 %), 2,025 involved specialized palliative medicine (28.7 %), and 3,516 involved palliative medicine consultation services (49.8 %, multiple coding possible). |
|  |  | (*b*) Report category boundaries when continuous variables were categorized |  | n/a |
|  |  | (*c*) If relevant, consider translating estimates of relative risk into absolute risk for a meaningful time period |  | n/a |

Continued on next page

| Other analyses | 17 | Report other analyses done—e.g., analyses of subgroups and interactions, and sensitivity analyses |  |  |
| --- | --- | --- | --- | --- |
| Discussion | | | | |
| Key results | 18 | Summarise key results with reference to study objectives | Page 11 | - Intensive care was required in 11.2% of all hospital cases, with long-term ventilation >95 hours needed in 0.8% of cases.  - The overall ICU mortality rate was 11.1%, rising to 38.8% for long-term ventilation cases, predominantly among patients aged ≥65 and males.  - Complex or specialized palliative care was provided in 53,875 ICU cases (0.8%) and 7,066 long-term ventilation cases (1.4%). |
| Limitations | 19 | Discuss limitations of the study, taking into account sources of potential bias or imprecision. Discuss both direction and magnitude of any potential bias | Page  12 | - data rely on billed hospital cases, which may not directly reflect the number of individual patients. Deceased patients are a special consideration, as they typically only experience one instance of death, thus aligning the number of hospital cases with the number of patients in this scenario.  - Data collected indicates the rate of ICU - and palliative care during the same hospital stay. No conclusion on temporal association between both treatments (parallel or sequentially) can be drawn. The "real rate" of specialized palliative medical care involvement in ICU patients is likely to be lower, since ICU physicians may provide basic palliative medical treatment themselves.  - Around 70 palliative care units in Germany are classified as "special facilities" (*besondere Einrichtungen*), meeting specific criteria and remunerating outside the DRG system. These cannot be accounted for in our dataset. Theoretically, patients could be discharged directly from ICU into Specialized Palliative Home Care (SAPV). Since the remuneration of outpatient services is not dealt with by the hospital remuneration system, these cases cannot be accounted for.  - Data collected observes statistically significant differences in ICU-related mortality and access to palliative care based on gender and age. These findings however do *not* imply a causal relationship, as critical potential confounding factors could not be analysed. Nonetheless, our findings are consistent with previous studies reporting similar associations.  - Data provided doesn’t cover key aspects in palliative care such as symptom burden, quality of life, and stress experienced by ICU patients and their families. Thus, the actual assessment of palliative care demand is impossible. Not all ICU patients require specialized palliative care. Basic palliative care might have been provided by ICU physicians.  - Data spans the duration of the SARS-CoV-2 pandemic in Germany, potentially influencing diagnoses and treatment outcomes. However, data from 2019 (pre-Covid pandemic) is consistent with data from the following pandemic years 2020-2022.  - Changes in healthcare policies, reimbursement practices, or guidelines during the study period were not taken into account. To our knowledge, no major changes in healthcare policies and reimbursement practices were implemented between 2019 and 2022. |
| Interpretation | 20 | Give a cautious overall interpretation of results considering objectives, limitations, multiplicity of analyses, results from similar studies, and other relevant evidence | Page 13 | Despite facing life-threatening illnesses and high mortality rates, only 0.8% of ICU patients and 1.4% of long-term ventilated patients received specialized palliative care. These real-world data highlight the current state of palliative care integration in ICU treatment. Efforts are needed to enhance access to palliative care for ICU patients and their families with complex needs. Implementing a benchmarking process could help achieve this goal. |
| Generalisability | 21 | Discuss the generalisability (external validity) of the study results |  | Data spans all billed cases over four years in Germany. It reflects the current state of integration of Palliative Care in ICU-Care in Germany. Since data was collected in Germany only, it cannot give Information on the state of integration in our countries, though studies suggest a similar picture. Introducing a benchmarking process could be helpful in implementing more Palliative Care into ICU-Care. However, a benchmark itself (how much Palliative Care is actually needed) must be established first. |
| Other information | |  | | |
| Funding | 22 | Give the source of funding and the role of the funders for the present study and, if applicable, for the original study on which the present article is based |  | No funding was used implementing this study. |

*Give information separately for cases and controls in case-control studies and, if applicable, for exposed and unexposed groups in cohort and cross-sectional studies.

**Note:** An Explanation and Elaboration article discusses each checklist item and gives methodological background and published examples of transparent reporting. The STROBE checklist is best used in conjunction with this article (freely available on the Web sites of PLoS Medicine at http://www.plosmedicine.org/, Annals of Internal Medicine at http://www.annals.org/, and Epidemiology at http://www.epidem.com/). Information on the STROBE Initiative is available at www.strobe-statement.org.
